# Numerical analysis of the impact of flow rate, heart rate, vessel geometry, and degree of stenosis on coronary hemodynamic indices

**DOI:** 10.1186/s12872-018-0865-6

**Published:** 2018-06-28

**Authors:** Zbigniew Malota, Jan Glowacki, Wojciech Sadowski, Marcin Kostur

**Affiliations:** 1 Biocybernetics Laboratory, Prof. Z. Religa Foundation of Cardiac Surgery Development, Wolnosci st. 345a, 41-800 Zabrze, Poland; 2 Department of Radiology, Silesian Medical University, 3-go Maja st. 13/15, 41-800 Zabrze, Poland; 3 Department of Diagnostic Imaging, Silesian Center for Heart Diseases, Curie-Skłodowskiej st. 9, 41-800 Zabrze, Poland; 4 Institute of Physics, University of Silesia, 75 Pułku Piechoty st. 1, 41-500 Chorzow, Poland; 5 Silesian Center for Education and Interdisciplinary Research, University of Silesia, 75 Pulku Piechoty st. 1, 41-500 Chorzow, Poland

**Keywords:** Coronary artery stenosis, Computational fluid dynamics, Hemodynamic indices

## Abstract

**Background:**

The stenosis of the coronary arteries is usually caused by atherosclerosis. Hemodynamic significance of patient-specific coronary stenoses and the risk of its progression may be assessed by comparing the hemodynamic effects induced by flow disorders. The present study shows how stenosis degree and variable flow conditions in coronary artery affect the oscillating shear index, residence time index, pressure drop coefficient and fractional flow reserve. We assume that changes in the hemodynamic indices in relation to variable flow conditions and geometries evaluated using the computational fluid dynamics may be an additional factor for a non-invasive assessment of the coronary stenosis detected on multi-slice computed tomography.

**Methods:**

The local-parametrised models of basic shapes of the vessels, such as straight section, bend, and bifurcation as well as the global-patient-specific models of left coronary artery were used for numerical simulation of flow in virtually reconstructed stenotic vessels. Calculations were carried out for vessels both without stenosis, and vessels of 10 to 95% stenosis. The flow rate varied within the range of 20 to 1000 ml/min, and heart rate frequency within the range of 30 to 210 cycles/min.

The computational fluid dynamics based on the finite elements method verified by the experimental measurements of the velocity profiles was used to analyse blood flow in the coronary arteries.

**Results:**

The results confirm our preliminary assumptions. There is significant variation in the coronary hemodynamic indices value caused by disturbed flow through stenosis in relation to variable flow conditions and geometry of vessels.

**Conclusion:**

Variations of selected hemodynamic indexes induced by change of flow rate, heart rate and vessel geometry, obtained during a non-invasive study, may assist in evaluating the risk of stenosis progression and in carrying out the assessment of the hemodynamic significance of coronary stenosis. However, for a more accurate assessment of the variability of indices and coronary stenosis severity both local (near the narrowing) and global (in side branches) studies should be used.

## Background

Coronary artery disease (CAD) is a major cause of death in economically developed countries [1]. CAD occurs when the coronary arteries become narrowed or blocked as a result of the build-up of fat, cholesterol and other substances within the artery wall. This process is called atherosclerosis.

The current gold standard for the assessment of severity of atherosclerotic lesions still remains invasive coronary angiography. However, the method has some limitations. One of them is the fact that two-dimensional images of the atherosclerotic plaque is different in different planes of the projection. In this case the visual assessment of stenosis in coronary angiography is subject to error and does not express the functional significance of the lesion [2]. In addition, not only the degree of narrowing but also the shape and location of the atherosclerotic plaque may indicate its hemodynamic significance. Therefore, some lesions that are considered hemodynamically insignificant may cause a reduction in coronary flow reserve (CFR). For these reasons, especially in the case of intermediate stenosis, in which the reduction of the arterial light is in the range of 50 to 70% (“borderline stenosis”), the fractional flow reserve (FFR) measurement should be performed [2, 3].

Computational fluid dynamics (CFD) technique may be used for the assessment of hemodynamic significance of lesions detected by coronary CT angiography (FFR_CT_) [4, 5]. However, the numerical simulation of blood flow through arteries under normal physiologic conditions is extremely difficult because of the complex anatomy of coronary vessels, the flexibility of the arterial wall, the pulsatile flow, the variable vascular resistance, and the non-Newtonian properties of blood. Therefore, it is difficult to develop an appropriate numerical model of coronary vessels that is fully consistent with the clinical data. Additionally, there is usually a deficit of clinical data required to develop a detailed numerical model of a coronary vessels.

For steady flows and pulsatile flows, Young and Tsai [6] demonstrated that drop in pressure in a narrowed vessel can be represented in a simplified way as a function of pressure gradient and flow rate. The function is a quadratic polynomial where the first element determines the effect of the viscosity forces and the second one is due to convective acceleration, which leads to flow separations and energy losses due to turbulence flow. In the case of pulsatile flow, there is also a third segment that is related to the activity of the inertial forces. The coefficients of this polynomial can only be determined empirically.

However, hemodynamic significance of patient-specific coronary stenoses and the risk of its progression may be assessed by comparing the hemodynamic effects induced by flow disturbances both in narrowed coronary vessel and computer-reconstructed coronary artery without stenosis.

Hence, we propose a simplified a non-invasive test based on a numerical modelling method. The method involves analysing selected hemodynamic indices such as the oscillating shear index (OSI), residence time index (RRT), pressure drop coefficient (CDP), and fractional flow reserve (FFR_CT_) in relation to variable flow conditions and variable geometries of coronary vessels. We assume that a non-linear change in these parameters induced by disturbed flow will facilitate the assessment of the hemodynamic significance of stenosis.

## Methods

Commercially available software Ansys 17.1 (ANSYS, Inc.) based on FEM (Finite Element Methods) was employed for blood flow simulation by numerical solution of the Navier-Stokes and continuity equations.

**Fig. 1:**
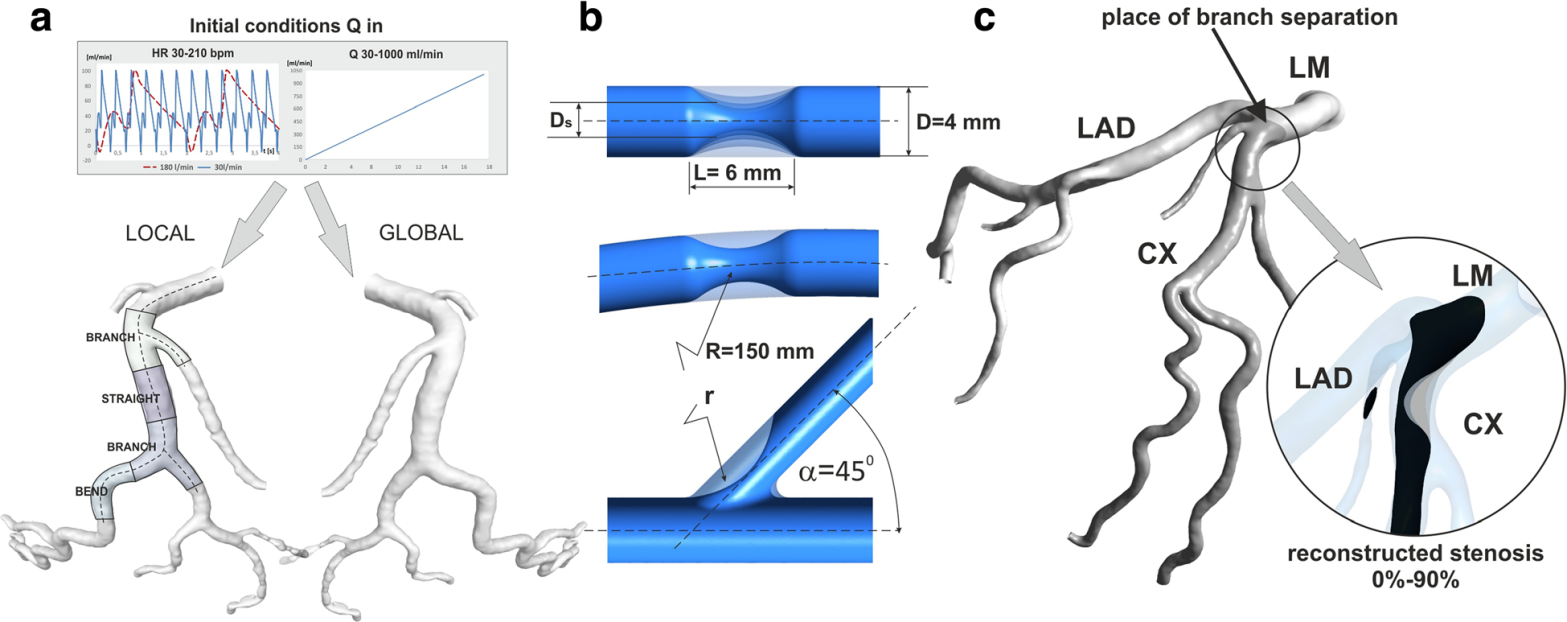
**a** Diagram of an initial condition for both local and global approach to computational study, **b** local-parametrised models of basic shape of the vessel: straight section, bend and a bifurcation, **c** global-patient-specific model of left coronary artery of patients with stenosis (~ 10%) in the proximal segment of the left circumflex (CX) branch and with the virtually reconstructed of coronary arteries, both without stenosis as well as 24, 62, 75 and 90% stenosis. LM- Left Main Artery, LAD- Left Anterior Descending Artery, CX- Left Circumflex Artery

The local blood flow characteristics are closely correlated with global hemodynamics of cardiovascular system. The certain local changes, like those of artery stenosis, can lead to a change in global blood flow distribution. Stenosis in one branch of coronary artery, can affect on the flow and pressure distribution in the other branches. For this reason, two types of coronary geometrical models were used (Fig. 1):

- **The parameterised models** - the axially symmetric vessels with basic shapes: straight section, bend and a bifurcation. The basic dimensions are shown in Fig. 1b. The narrowing was placed at a distance of 200 mm from the inlet. The calculations were performed for both vessels without stenosis and vessels with 10 to 95% stenosis.
- **The patient-specific models** - the left coronary artery with stenosis (~ 10%) in the proximal segment of the left circumflex branch (CX). The 3D geometry of the patient-specific models were established using DICOM image segmentation in the 3D-DOCTOR software package (Able Software Corp.). Numerical reconstruction of arteries, both without stenosis as well as with 24, 62, 75 and 90% stenosis was also performed. The coronary models were divided into two areas: an area where narrowing occurs (CX) and an area where there is no stenosis (left anterior descending artery (LAD). This makes it possible to analyse the influence of the degree of coronary stenosis located in the CX artery on the hemodynamic indices in the other branches of the left coronary artery (Fig. 1c).

Our numerical simulation is based only on non-invasive coronary imaging techniques. The personalized clinical data regarding both flow and pressure conditions, rheological blood properties as well as mechanical properties of the arterial walls and atherosclerosis plaques usually are missing. For this reason, and because of the need to verify the simulation results on a hydraulic blood circulation system, the numerical model was simplified. The key assumptions of the simulation model are presented below:

- Inflow boundary
  o Mass flow boundary conditions was used in ANSYS CFX to provide a prescribed flow rate and mass flux distribution at an inlet of coronary artery (Fig. 1a).
  o Flow directions was specified as normal to the inlet boundary.
  o For the steady flow, the mass flow rate was increased linearly with time from 30 to 1000 ml/min (average Reynolds values of 60 to 2240).
- For the pulsatile flow, waveform of pulse was appropriate for the inlet left coronary artery. The frequency of heart rate was variable from 30 to 210 bpm and amplitude was increased linearly from 100 to 200 ml/min. The stroke volume (SV) remained constant i.e. was independent of heart rate and autoregulation.
- Outflow boundary
  o Open boundary condition was used for all outlet of the coronary vessels (Entrainment option with relative static pressure equal zero was chosen in ANSYS CFX setup).
- Wall boundary
  o The rigid vessel, no-slip, no moving, the velocity of the fluid at the wall boundary was set to zero.
- Fluid model
  o Newtonian, incompressible, no mesh deformation with dynamic viscosity μ = 3.4 cP and density ρ =1056 kgm^− 3^.
- Analysis type
  o Laminar, steady and transient flow with at least 3 heart cardiac cycles, (the 3-rd cardiac cycle was analysed), time steps 0.001 s for steady flow and 0.001 s*60/HR for pulsatile flow.
- Solver settings
  o High-resolution advection scheme, Second Order Backward Euler transient scheme with RMS (root mean square) residual level of 1e-5.
- Mesh of FEM
  o Tetrahedral mesh generation, editing and diagnostic were carried out using ANSYS ICEM CFD.
  o The mesh of the FEM contained approximately 900,000 node elements and around the wall, branches and narrowing, was thickened and adapted for the CFX Ansys solver (patch conforming).

The impacts of the flow rate, heart rate, vessel geometry, and degree of stenosis on the following diagnostic indices were assessed:

- **Pressure drop (ΔP)** at the vessel, the function of viscous losses, linearly correlated with the flow rate and losses due to momentum changes, and it varies with the second power of the flow rate.
- **Pressure drop coefficient (CDP)** [7, 8], the zero-dimensional index that which takes into account the pressure drop in the narrowing and the proximal flow velocity U (spatial and temporal mean). It expresses the ratio of the pressure drop caused by the stenosis to the dynamics pressure:
  1 $$ CDP=\frac{\Delta p}{0.5{\rho U}^2} $$

where Δp is composed of the viscous pressure losses and losses related to momentum changes.

- **Wall shear stress (WSS)** [9], the tangential frictional force on the endothelial surface**.**
  For a Newtonian fluid flow of in the straight vessel the shear stress is proportional to the flow shear, and WSS can be expressed as:
  2 $$ WSS={\left.\mu \frac{\partial u}{\partial n}\right|}_{wall} $$
  where μ is the dynamic viscosity, u is the flow velocity parallel to the wall.
- **Oscillatory shear index (OSI)** [10] defined based on the temporal fluctuation of the WSS as:
  3 $$ OSI=\frac{1}{2}\left(1-\frac{\left|\underset{0}{\overset{T}{\int }}\overline{WSS} dt\right|}{\underset{0}{\overset{T}{\int }}\left|\overline{WSS}\right| dt}\right) $$
  where *WSS* [Pa] is the magnitude of the temporary local wall shear stress.
- **Relative residence time (RRT)** [9] defined as:
  4 $$ RRT={\left[\left(1-{2}^{\ast } OSI\right) AWWS\right]}^{-1} $$

where $$ AWSS=\frac{1}{T}\underset{0}{\overset{T}{\int }}\mid \overline{WSS}\mid dt $$_._

- **Fractional flow reserve (FFR)** [5], the ratio of the maximum coronary flow in the presence of a stenosis to the maximum flow in a vessel without stenosis. It can be determined as the ratio of the average pressure measured after the stenosis (P_d_) in the distal section of the vessel to the average pressure in the aorta (P_a_) in the conditions of maximum hyperaemia (excess of blood):
  5 $$ FFR=\frac{Qs}{Qn}=\frac{\left({P}_d-{P}_v\right)/R}{\left({P}_a-{P}_v\right)/R}=\frac{P_d}{P_a} $$
  where *P*_*a*_ is the pressure in the aorta, *P*_*v*_ is the central venous pressure, and *P*_*d*_ is the pressure after the stenosis.
  Non-invasive Fractional Flow Reserve derived from coronary CT (FFR_CT_) for pulsatile flow is calculated as the time averaged pressure (at each node of fluid) to time averaged Inlet pressure ratio.

### The experimental verification of the numerical models

To verify the numerical model, a precise analysis of the velocity profiles was performed on a hydraulic blood circulation system based on the Windkessel effect [11]. The dynamics test was based on measurements of the flow rate, pressure, and velocity profiles obtained using an impulse ultrasonic flowmeter [12] in the model of an axially symmetric vessel with 75% stenosis. The model was rescaled 4 times while maintaining geometric, kinematic, and dynamic criterion of similarity.

**Fig. 2:**
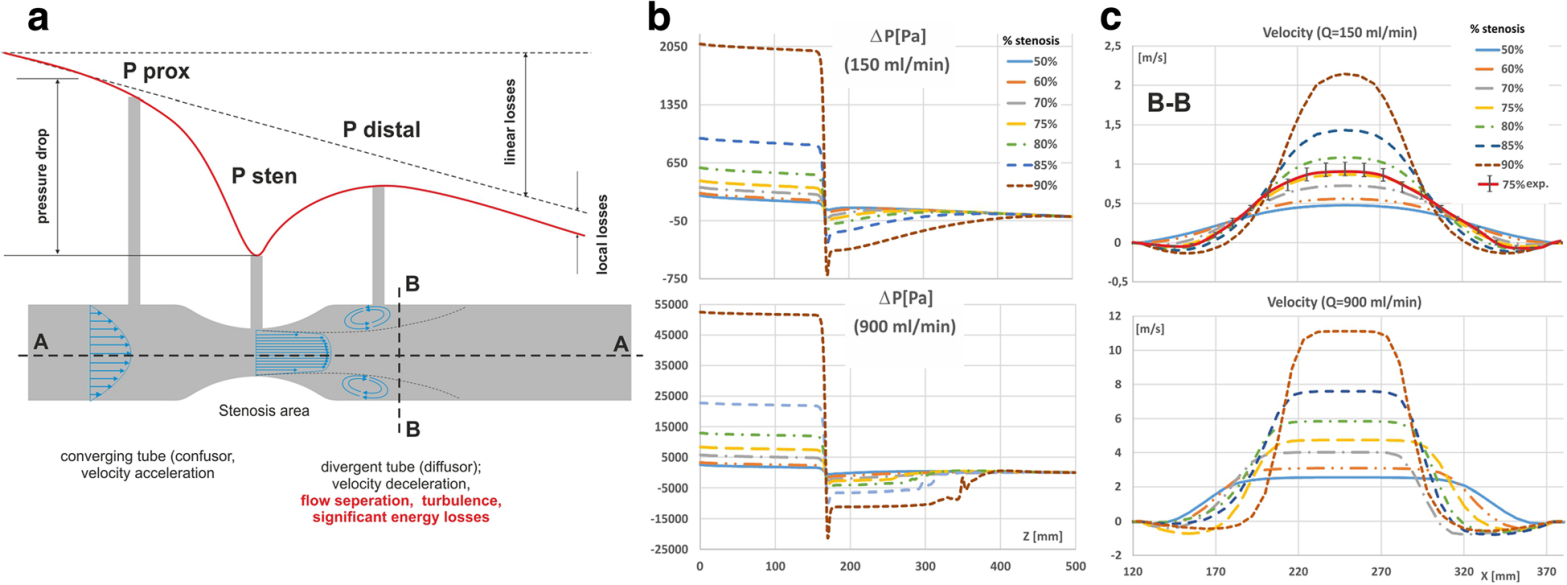
The flow through a stenotic tube; a) division of the stenosis area into: converging, narrowing and (**a**) diverging section, each with their particular pressure-flow relations, **b** pressure drop along a length of narrowed vessel, **c** velocity profiles, 12 mm behind the stenosis, for different degrees of stenosis and flow rates

The flow profiles at a distance of 120 mm before the stenosis and 12 mm behind the stenosis were compared for different Reynolds numbers (Fig. 2c). The maximum Reynolds numbers in the tests did not exceed 2240. Nevertheless, critical Reynolds numbers are strongly correlated with the shape and size of the stenosis and the HR [13]. Recent research demonstrated that the fully turbulent nature of the flow occurs in cases of more than 50% stenosis [14]. However, our tests performed on the physical models demonstrated that for a 75% stenosis and low Reynolds numbers, the flow velocity profile obtained from a physical stand is the closest to the laminar model.

The main goal of our study is to compare hemodynamic parameters with variable flow conditions, geometries of vessel, and the degree of stenosis. Therefore, it was impossible to select one appropriate model for the entire scope of the test. For this reason, we decided to apply laminar-flow Newtonian blood models based on verification tests in our first studies. Many of the numerous publications devoted to the simulation of flow through stenosis in the coronary vessels also contain tests on laminar Newtonian models [15].

## Results

### Velocity distribution

In vessel stenosis the shear stresses increase, the velocity profile widens, and the viscous boundary layer becomes very thin. Downstream to the narrowing, there is a negative flow in a separated swirl layer (Fig. 2a). High stresses appear at the boundary of the main jet between the main stream and the vortex area. The separation area depends on the geometry of the stenosis and Reynolds number.

Further from the stenosis, there is an enhanced boundary layer with a low velocity gradient. An axially non-symmetric stenosis, such as one in the internal curvature of the bend of a vessel, introduces additional disturbances to the flow in the plane that is perpendicular to the axis of the vessel. The shape of the velocity profile depends on the degree of stenosis. The velocity profiles at a distance of 12 mm behind the stenosis have a laminar character with a clear reversed flow in the area of the vascular wall. For higher flow intensity, the velocity profile has a ‘plug’ shape that is not fully developed and has a constant velocity in the central part of the vessel as well as high shear velocities and a thicker boundary layer (Fig. 2c). For very high flow rates, the velocity profiles are no longer symmetrical due to secondary flow perpendicular to the axis of the vessel.

### Vessel geometry

**Fig. 3:**
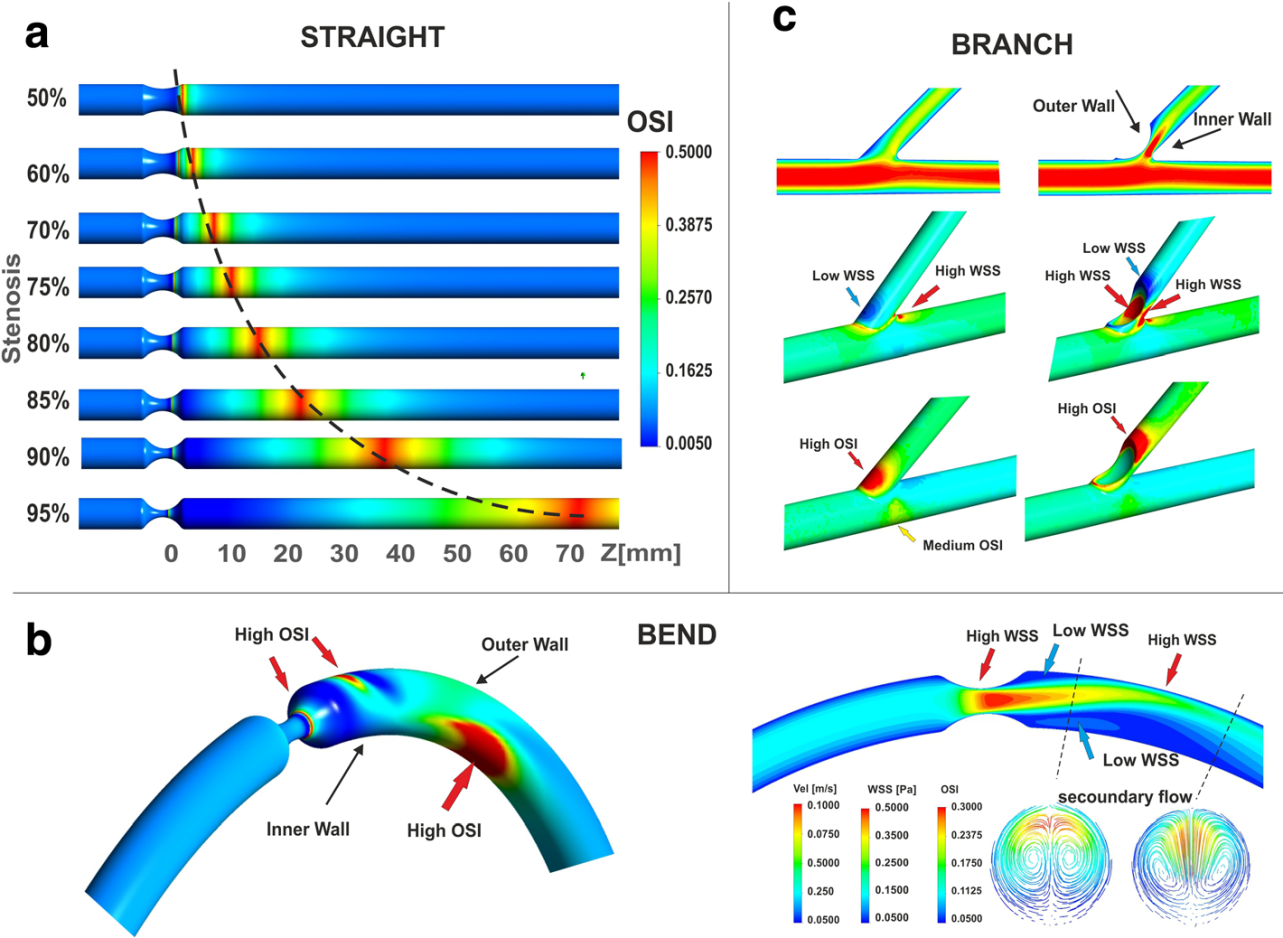
The effect of the vessel geometry on the distribution of the extreme values of OSI and WSS in: **a** straight vessel, **b** bend of the vessel, **c** branch of the vessel

The bend of coronary vessels (Fig. 3b) have a significant effect on the flow disturbances. Additional complexity of the blood flow in the bend results from the particle movement in the vessel not being parallel to the bent flow axis. The core of the flow (non-viscous in approximation) is well described by Bernoulli’s equation.

The pressure increases from the axis of the vessel in the radial direction toward the outside. The difference in these pressures is balanced by centrifugal forces and the activity of viscosity forces. Thus, a secondary flow is created in the section that is perpendicular to the axis of the vessel, which influences the distribution of the longitudinal velocity (Fig. 3b), and the flow stream in the bend area is spiral [16].

A smaller radius of the arch or higher flow velocities at the inlet results in more twisted stream helixes (with smaller spiral pitch), as well as higher kinetic energy losses. If the radius of the vessel arch is too small, places with stream detachments occur, which increase local flow losses. On the other hand, if the bend is slight, stream detachments do not occur, but the flow losses grow due to the friction forces resulting from the increased area of the channel.

In contrast to the straight section of the vessel, there are two areas with low values of WSS (high OSI). The first directly downstream to the stenosis on the outer wall of artery bending, the second farther away from the narrowing, on the inner wall (Fig. 3b).

Two areas with minimal WSS (high OSI) values also appear in the vessel branching. However, the first one is located in the side branch downstream of the stenosis on the outer wall, while the second one occurs on the wall in the main branch, below the place where main stream is slightly deflected. At the area where a vessel branches off, the velocity profile is not symmetrical.

Flow separation and stagnation point are observed in place, where the main flow stream is divided by branch apex. This point is area with no flow that is characterized by a near-zero pressure gradients and low WSS.

As you can see, the branching of the vessel also has a major influence on the blood flow distribution and thus on the hemodynamic indices and mechanical properties of vessel wall [17]. However, it should be noted that, assuming constant vascular resistance (setting aside of coronary autoregulation), an increase of the degree of stenosis in one branch causes a decrease of the flow rate in that branch, as well as a simultaneous increase of the flow rate in the branch with an unchanged cross section. This implies that the total pressure drop and wall stresses in the entire bifurcation are much lower than in cases of straight axially symmetric vessels and arches.

### Pressure drop

**Fig. 4:**
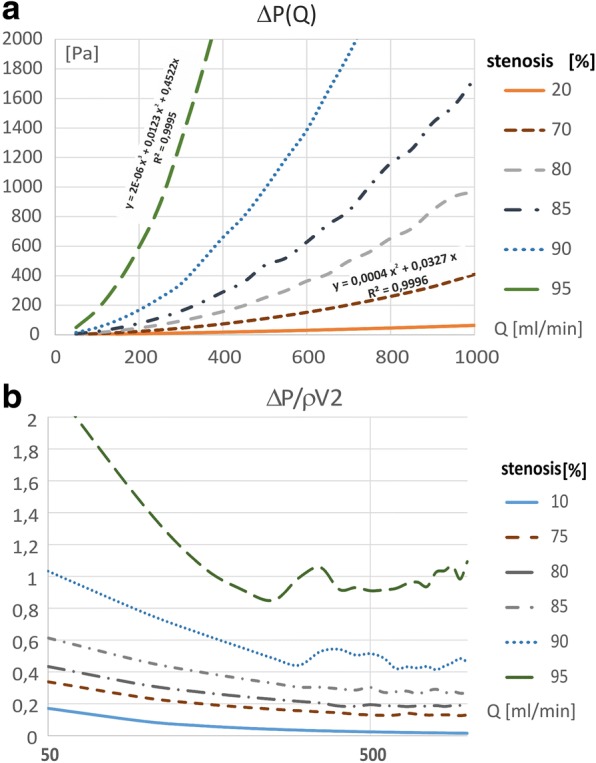
**a** ΔP, **b** CDP, as a function of the flow rate and the stenosis degree, for the straight models

When blood passes through the stenosis results in an additional, local pressure drop according to Bernoulli’s equation for real liquids (Fig. 2a), [18]. The pressure drop increases with the flow rate (Fig. 2b, Fig. 4a). In general, the increase of the pressure gradient can be described by means of a quadratic polynomial [19]. However, for high stenosis degree and high flow rate this relationship shows deviations from this quadratic function. For example, in cases of 70% stenosis, the clear deviation from quadratic plot occurs at a flow rate of around 900 ml/min, whereas when the stenosis is only 5% greater (75%), the disturbance occurs at 700 ml/min. In the case of stenosis above 95%, the pressure gradient-flow rate relationship is better described by a third-order polynomial with R-squared of 0.9995.

### Pressure drop coefficient

The CDP coefficient for the steady flow presents the flow resistance resulting from the activity of the viscosity forces and the change of the momentum according to eq. (1). The CDP and flow rate relationship has a clear local minimum (Fig. 4b) related to the transitional flow where the effects of turbulence become increasingly important. For higher flow rate, the CDP becomes practically independent of the flow rate with slight fluctuations that depend on both the degree of narrowing and the flow rate. CDP also increases non-linearly as the degree of stenosis increase. The greater the narrowing, the greater the increase in CDP.

### Wall shear stress

**Fig. 5:**
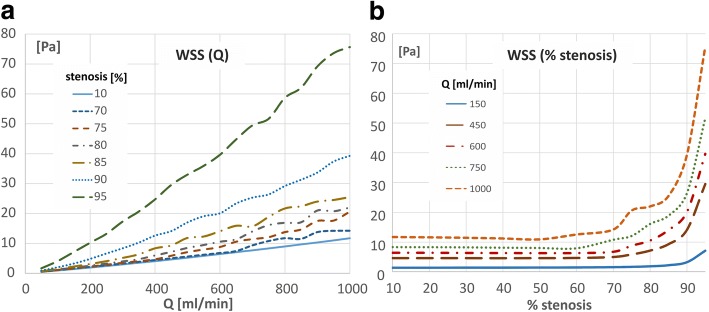
WSS as a function of: **a** the flow rate, **b** the stenosis degree for the straight, local-parametrised models

Generally, in stenosed artery, for both local and global models, the average WSS increases with the flow rate. For a straight model of the artery with up to 50% vessel stenosis, the WSS in functions of flow rate increases linearly (Fig. 5a). In case of higher values of stenosis degree this function is not linear. The higher the degree of stenosis, the earlier the deviation from linearity occurs. For 70% stenosis, this deviation is clear with flow rate beyond 650 ml/min but for 90% stenosis this deviation already occurs at the flow rate ~ 200 ml/min.

However, the WSS in function of stenosis degree is constant, up to a critical value of narrowing that depends on the flow rate, above which there is a significant increase of WSS (Fig. 5b). The higher the flow rate, the earlier the increase occurs. For 150 ml/min this increase occurs at 87% stenosis but for 450 ml/min occurs near of 70% stenosis already.

The degree of stenosis and heart rates also has a very strong impact on the level and spatial distribution patterns of WSS. In general, the maximum WSS values occur in vascular wall inside the stenosis. In addition to this, the local extreme values of WSS strongly depends on artery geometry.

In the curved vessel, the maximum WSS occur downstream to the stenosis where the main jet impinges on the outer wall (Fig. 3b). The minimum WSS occur both directly downstream to the stenosis on the outer curvature of artery and on the inner wall below the place where the main flow stream is moving in direction of the outer wall, below zone of maximum WSS.

**Fig. 6:**
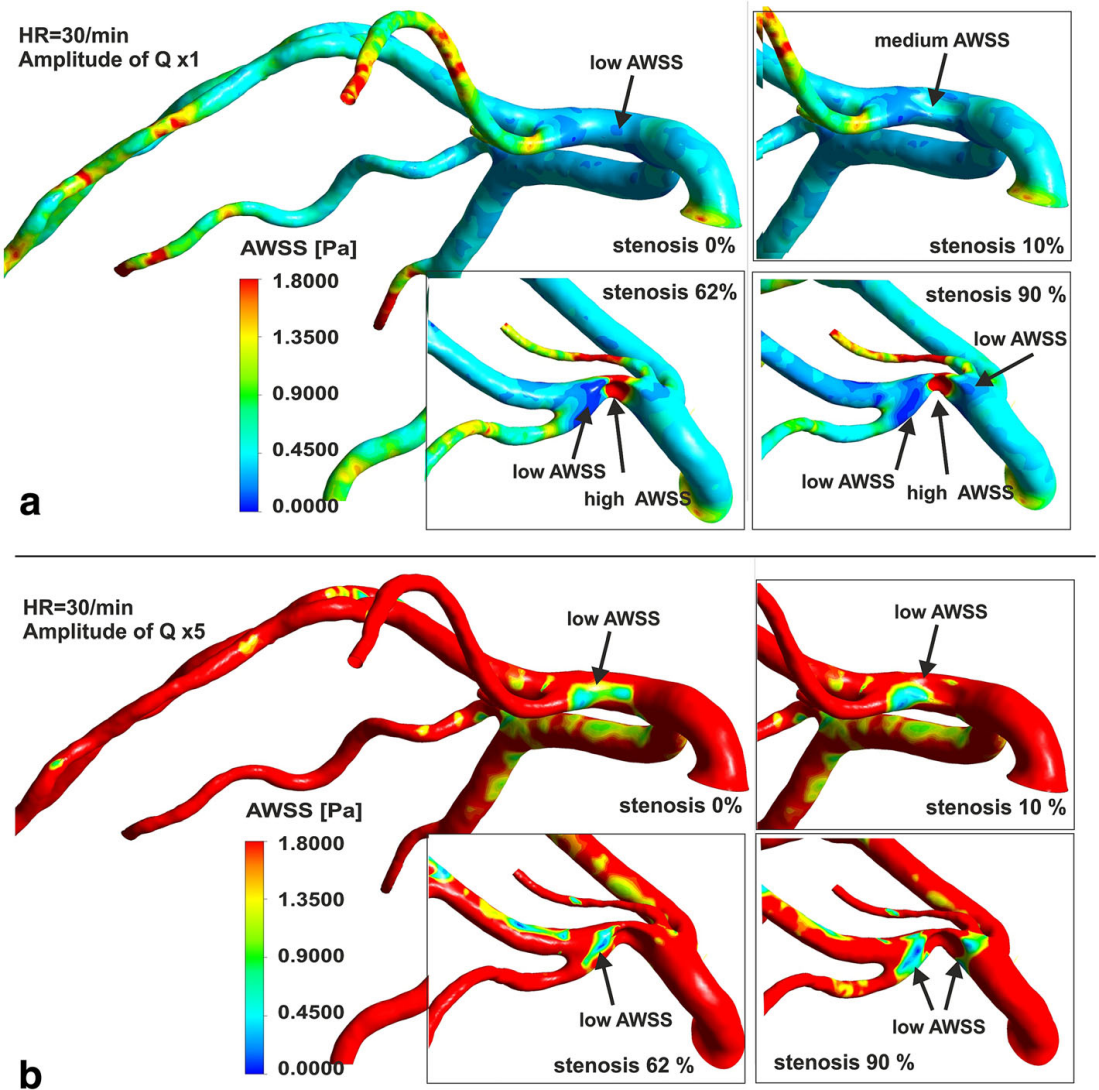
The effect of degree of stenosis and amplitude of pulsatile flow on AWSS index: **a** amplitude of Q x 1, **b** amplitude of Q x 5

In the case of vessel branching, wall shear level is observed to be much lower on the inner lateral wall than on the outer wall. WSS zone is observed on the inner walls near the flow divider tip (Fig. 5c). On the other hand, zone a very low WSS is present on the lateral outer wall, directly behind the branching, close to flow separation layer. The additional region of low WSS values occurs along the outer wall of main branch, downstream to the branch, near area of main jet deflection. Increasing the amplitude of pulsatile flow rate results in an increase in the mean and maximum AWSS values (Fig. 6). The 2-fold increase in amplitude increases 2-fold mean value of AWSS. The additional region of low WSS values occurs along the outer wall of main branch, behind the branch, near area of main jet deflection.

The effect of heart rate on the average of the WSSs is practically imperceptible.

### Oscillatory shear index and relative residence time

**Fig. 7:**
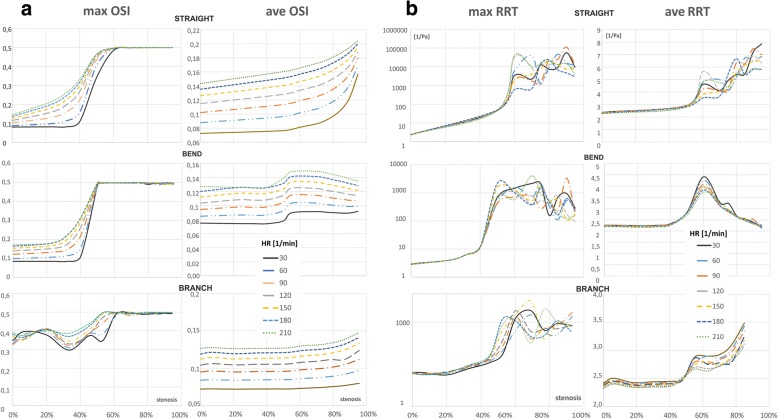
The influence of stenosis degree and the heart rate on the average and maximum value of: **a** OSI, **b** RRT, for local-parametrised models of the vessel: straight section, bend and a bifurcation

The OSI and RRT reveals the overall WSS oscillation during the pulsatile flow. An increase in heart rate frequency causes an increase OSI and RTT mainly influenced by the shorter duration of the cardiac cycle and temporal fluctuation of the WSS. Besides the heart rate frequency the OSI and RRT are affected by the degree of vessel stenosis as well. With up to 30–40% stenosis, the surface average value of maximum OSI is between 0.1 and 0.2, and its increases with the heart rate are practically linearly. Between 40 and 60% stenosis, there is a sudden increase in OSI to the maximum value of 0.5 (Fig. 7a). The higher heart rate, the slower increase in OSI. For the straight vessel, the mean OSI increases with the HR frequency and degree of vessel stenosis (Fig. 7). The relationship between RRT and the heart rate is more complex. Initially, the mean RRT is almost independent of the pulse frequency, and it increases linearly with the degree of stenosis. Above 50% stenosis there are sudden increases in RRT. Changing the vessel geometry has a strong influence on the relationship between studied indices and both the heart rate and degree of vessel narrowing.

The bends and branches of vessel cause the average values of both the OSI and RRT to be practically independent of the degree of stenosis. However, an abrupt increase of the OSI and RRT values are observed with degree of stenosis about 50–60%.

**Fig. 8:**
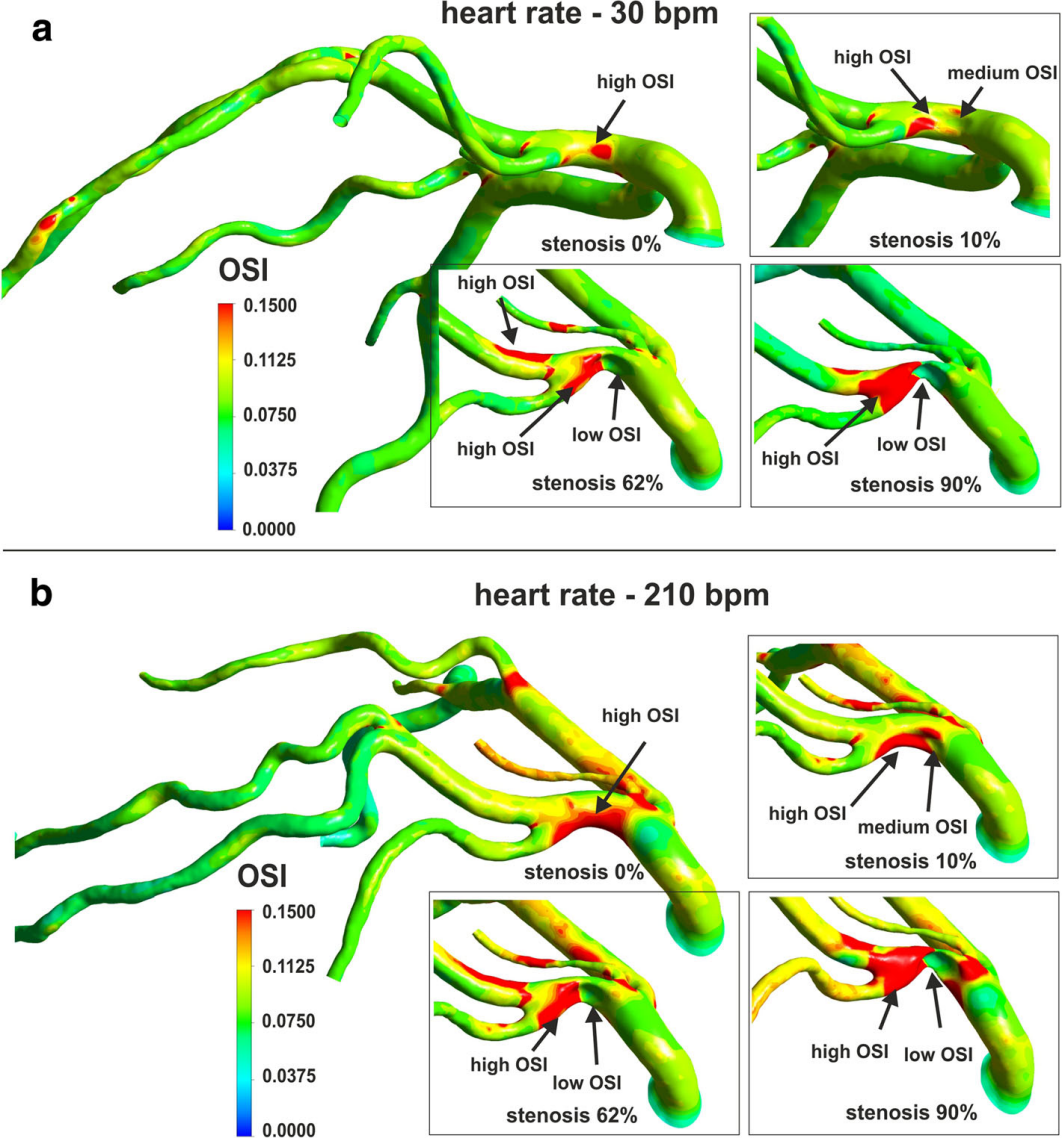
The effect of stenosis degree and heart rate frequency on OSI, for global-patient-specific model: **a** heart rate of 30 bpm, **b** heart rate of 210 bpm

**Fig. 9:**
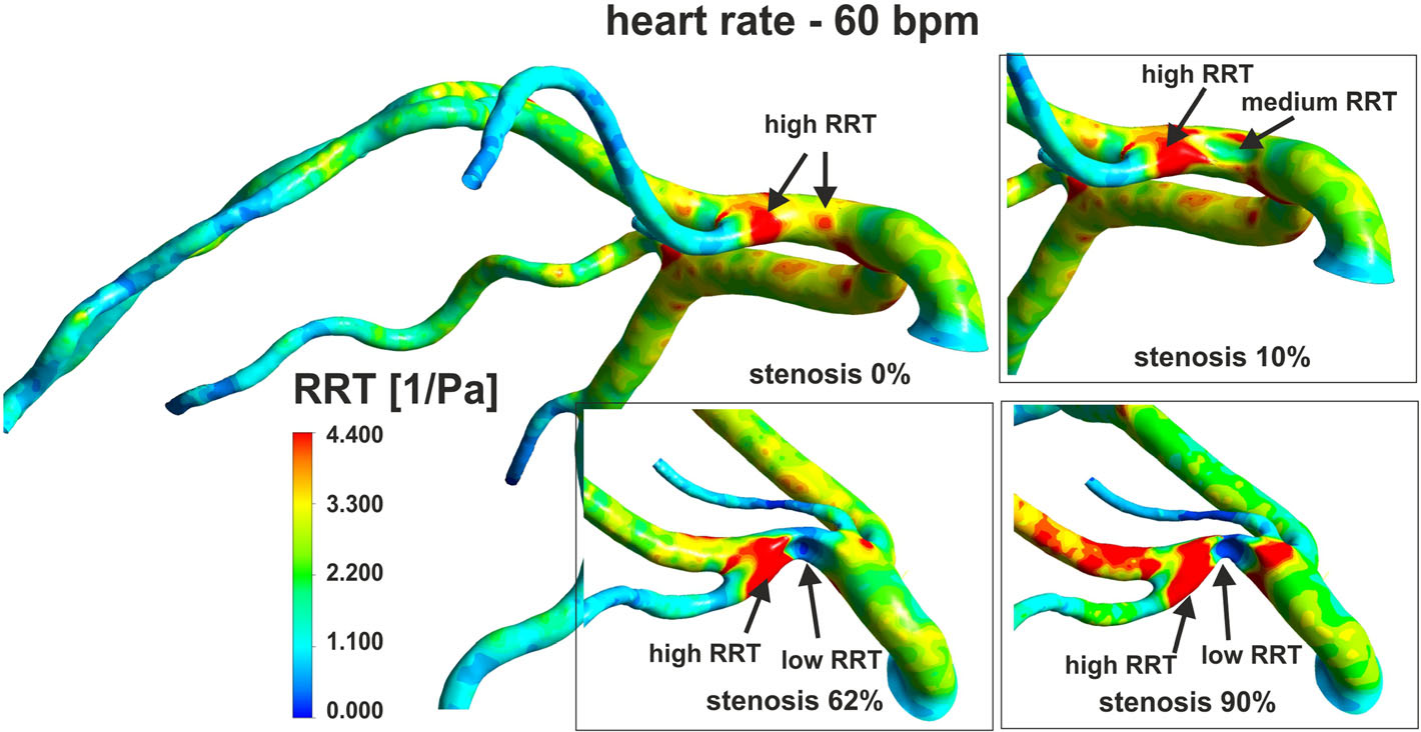
The effect of stenosis degree on RRT, for global-patient-specific model

The degree of stenosis also have a significant impact on the spatial distribution with extreme values of OSI and RRT (Fig. 8, Fig. 9). The maximum OSI and RRT occur in an area downstream from the stenosis where the minimum values of WSS appears. The highest OSI values is located immediately downstream of the stenosis on the vessel wall near the zones of vortex flow.

Along with the increase of the degree of stenosis, the distance from the region of maximum OSI and RRT to the stenosis position increases, irrespective of HR. In a straight section of vessel, this distance from the region of maximum OSI and RRT to the stenosis is a power function of the stenosis degree independent of HR (Fig. 3a). This “creeping RRT” (as well as “creeping OSI”) effect can lead to atherosclerosis plaque progression, increased flow resistance, and reduce the vessel’s cross sectional area (Fig. 3a). This effect was observed for both simple locally-parameterised models and the patient-specific model of the left coronary artery (Figs. 3, 8 and 9).

Generally, the higher the degree of stenosis, the further from the stenosis the area of maximum OSI and RRT occurs. In these locations, very low WSS values simultaneously occur, which are caused by the helical flow of the stream flowing out from the stenosis.

**Fig. 10:**
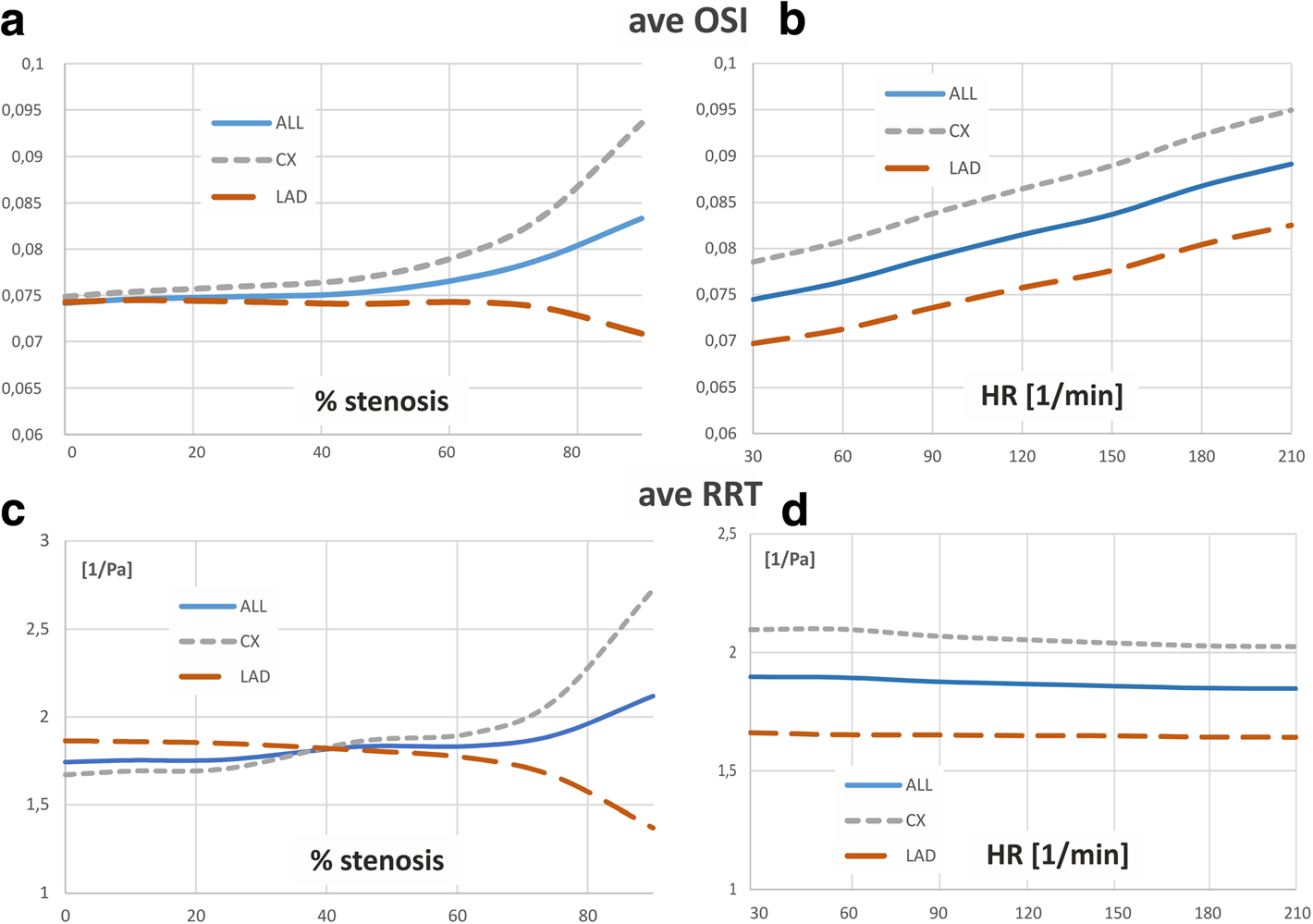
Effect of the: **a**, **c** stenosis degree, **b**, **d** heart rate frequency on the OSI and RRT for global-patient-specific model divided into two main branches: LAD (without stenosis) and CX artery (with stenosis)

The surface average OSI increases linearly with the heart rate. However, with up to 10% narrowing of the CX branch, the mean OSI values practically do not change (Fig. 10). A significant impact of the degree of stenosis on the average OSI is observed above 50% stenosis. When the degree of the stenosis increases in CX branch, the average OSI and RRT in these branches also increases. However, in LAD with stenosis, OSI and RRT decreases. Whereas, an increase of the HR frequency causes a linear increase of OSI in all branches, but the mean RRT is practically independent of the HR frequency (Fig. 10b, d).

### Fractional flow reserve

**Fig. 11:**
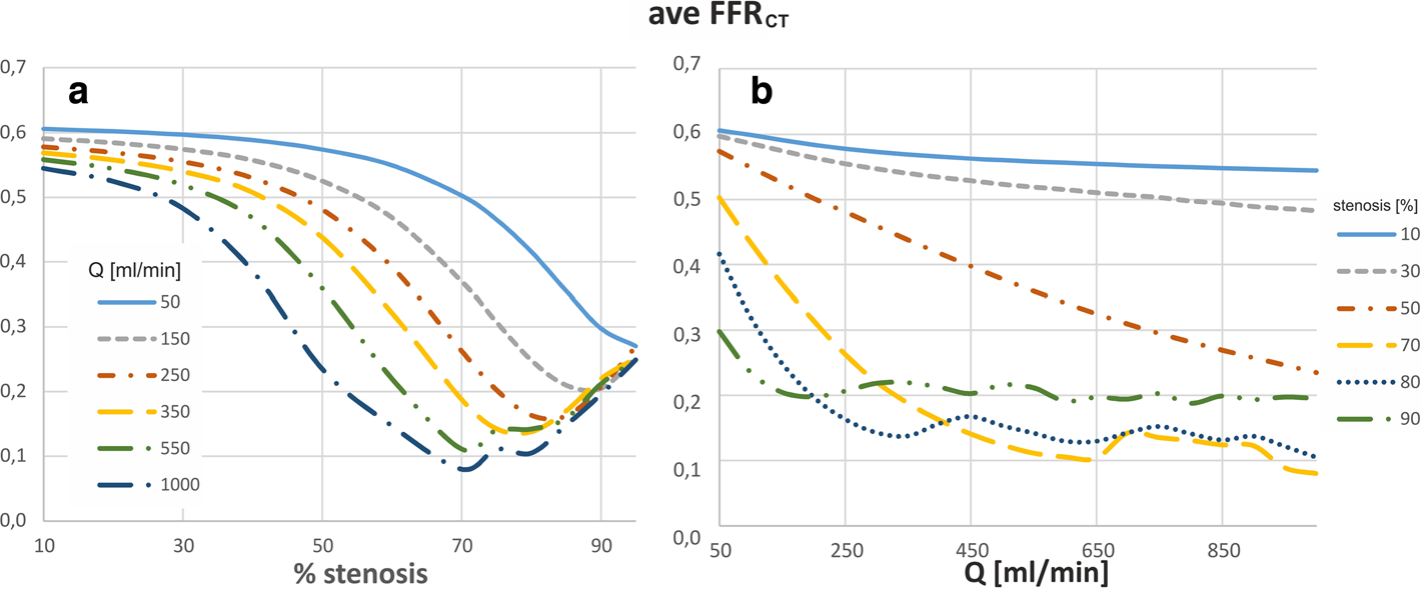
The influences of: **a** stenosis degree, **b** flow rate on the FFR_CT_ for local-parametrised models of straight section of vessel

FFR_CT_ across the stenosis in straight section of vessel significantly decreases with increases in both the degree of stenosis and flow rate (Fig. 11). However, at flow rates above 200 ml/min for narrowing greater than 70% the FFR_CT_ rises and becomes practically independent of the flow rate. For example, for 90% stenosis with flow rate above of 200 ml/min, the mean FFR_CT_ is constant value at ~ 0.2, but for 70% stenosis, the mean FFR_CT_ has a constant average value of 0.1 at flow rates above 650 ml/min.

**Fig. 12:**
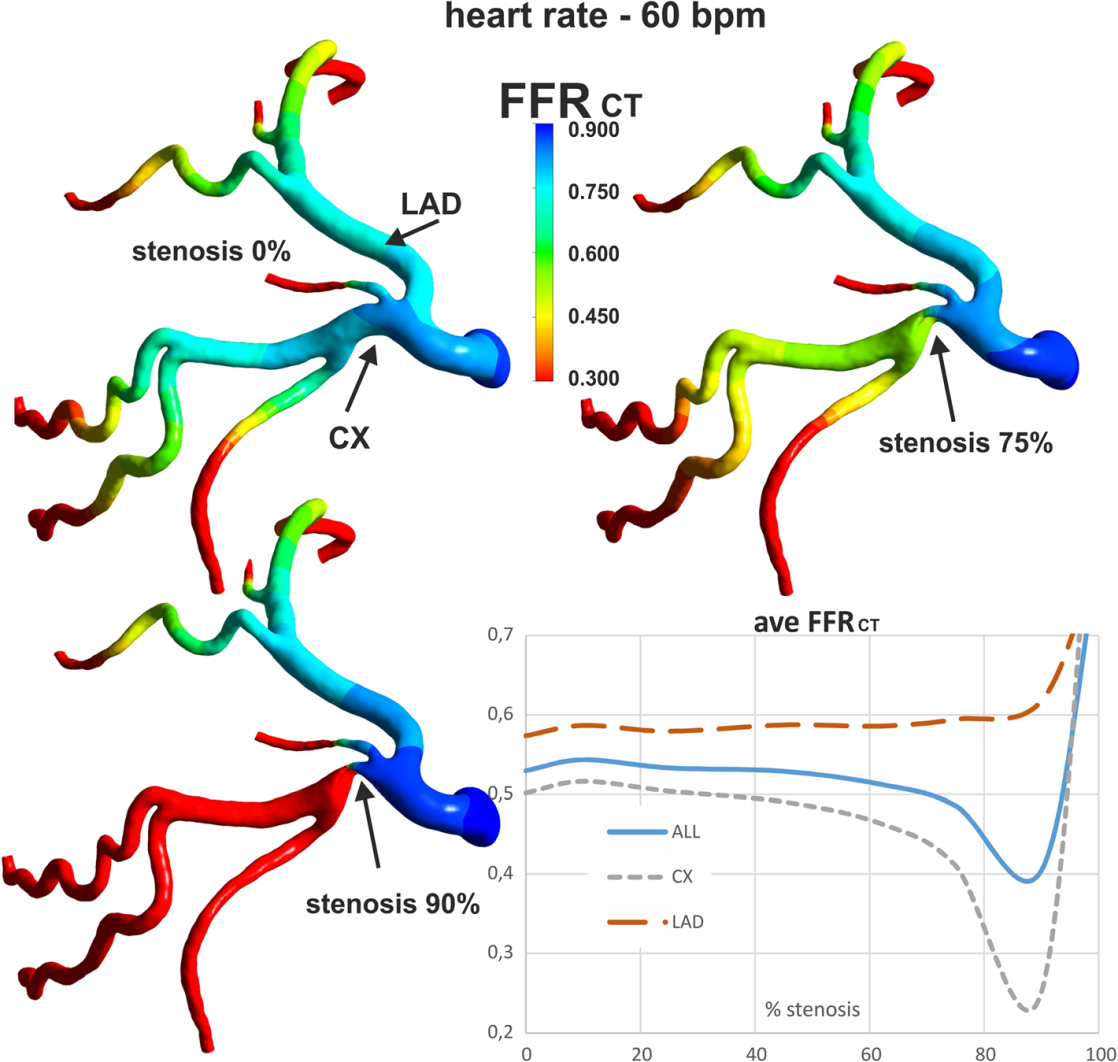
The effect of degree of stenosis on FFR_CT_ for patient-specific model of left coronary artery, divided into two main branches: LAD (without stenosis) and CX artery (with stenosis), for pulsatile flow

For the patient-specific model with low degrees of stenosis, the time and surface average FFR_CT_ initially minimally increases until 10% stenosis and then slowly decrease. At 70% stenosis, the FFR_CT_ decrease more rapidly. The FFR_CT_ decrease is observed only in the CX branch, downstream of stenosis (Fig. 12). The total occlusion of the CX branch (resulting in no flow) leads to an abrupt increase in the average FFR_CT_ in other branches to 0.9. The surface and time average FFR_CT_ is independent of the heart rate.

## Discussion

It is clear that coronary geometries and thus the flow patterns in arteries are very complex, involving secondary flow, flow separation and regions of high and low wall shear stress. The pattern distribution of velocity flow, pressure, WSS may have important implications for the distribution of atherosclerosis [20].

The simulation of flow both in parametrised and patient-specific models with simple geometry allowed analysis of both local and global flow losses. The global flow losses in the blood vessel system are caused by the activity of viscosity forces, which are proportional to the length of the artery. The local losses are related to the flow disturbance such as whirls, flow seperation, and secondary flows, naturally occurrence in the bends, narrowings, or bifurcations of the vessel [21]. The complex nature of blood flow in the area of abrupt stenosis of a blood vessel is connected with the need to determine the actual values of the local loss coefficients empirically and to determine their dependence on the hydrodynamic flow conditions.

It is extremely difficult to develop a proper numerical model of coronary vessels that are absolutely consistent with the clinical data. This is predominantly due to the complex geometry, vessel compliance, pulsatile flow, variable vascular resistance in the cycle, and non-Newtonian properties of blood. Other issues are the lack of detailed clinical hemodynamic data and the diverse physiologies of the circulatory system for each patient. Nevertheless, complex parameters can be analysed in a much broader scope due to the recent developments of diagnostic methods based on medical images, computer-based methods, and improved efficiency of numerical calculations. Thus, it is now possible to perform CFD analysis of the blood flow with variable flow conditions of parameterised models of complex circulatory systems in a short time.

### Flow rate

During the simulation, the inlet flow changes for both constant and pulsatile flow. In the case of a constant flow, the flow rate increases linearly. In the case of a pulsed flow, the change in the flow rate was caused by a change in the amplitude of the pulse. It should also be noted that the volumetric flow rate (cardiac output) increases as a result of an increase in heart rate, but this does not affect the flow rate during one cardiac cycle. The stroke volume in our study is constant.

According to the Poisseuille’s formula, flow rate in the vessel is proportional to the fourth power of its radius. Therefore, very subtle changes in vascular diameter have a profound impact on blood flow and the autoregulation is more effective in vessels without atherosclerosis, with more flexible walls [22]. Without taking into account autoregulation in numerical simulation, for the vessel without stenosis, the increase in the flow rate causes a linear increase in the pressure gradient.

However, the flow disturbance induced by stenosis causes a deviation from this law. The appearance of the vessel narrowing causes an additional pressure drop and the pressure gradient flow-rate relationship can be described by means of a quadratic polynomial [6]. In the case of vessel with stenosis greater than 90%, an increase in the pressure gradient takes place much faster and therefore, this relationship is better described by means of higher order polynomial.

However, the increase of flow rate cause a decrease in pressure drop coefficient. For higher flow rate, the CDP becomes practically independent of the flow rate. The point at which this occurs depends on the degree of stenosis and is pronounced only at higher stenosis values, so it can be a parameter facilitating the determination of significant stenosis.

It should be noted that increase in flow rate or in amplitude of pulsatile flow rate results in increases in the AWSS values. For this reason, the region of low AWSS values is much more pronounced and the analysis of the places, where the atherosclerosis plaque progression may occur, is more precise. That is why, it seems that, the assessment of the risk of stenosis progression and their hemodynamic significance should be performed at high flow rates or high amplitudes of pulsation.

### Heart rate

The unsteady flow phenomena play a major role in the blood flow through coronary arteries. In general, the pulsatile flow is characterized by complex spatial and temporal velocity distributions and reversed flow regions.

The increase in heart rate increases the number of cardiac cycles and thus increases of cardiac output [22]. However, it should be noted, that practically the heart stroke volume is decreased at higher heart rate. Therefore heart rate remains the most practical indicator of the heart’s work and the functions of cardiovascular system during exercise stress test.

In our virtual cardiac test, an increase of the heart rate frequency causes an increase of the cardiac output, but it does not cause any increase in the stroke volume (Fig. 1). Therefore, the values of OSI and RRT are influenced by inertial forces resulting from the shortening of the cardiac cycle and the increasing in both blood acceleration and temporal fluctuation of the WSS.

Low-density cholesterol and blood pressure may contribute to the formation and development of atherosclerosis. Probably, heart rate (HR) also can affect the low values of WSS and high values of OSI [22]. The highest values of OSI are observed for both high heart rate frequency and high amplitude of pulsatile flow rate in contrast to RRT. However, it should be noted that the value of OSI is more sensitive to the heart rate frequency and amplitude than RRT.

The spatial average OSI increase when increase in HR and reach the maximum value of 0.5 at 60% narrowing, practically independently of HR.

An increase in HR increases only the area of maximal OSI values in all branches of the coronary vessel. This parameter for high HR shows the places of a possible narrowing. However, it is difficult to determine the significance of the already existing narrowing only on the basis of OSI.

In contrast, RRT is independent of HR for low degree of narrowing. Above 60% of the degree of stenosis, there is a clear dependence on HR, which is very complex and difficult to clearly define.

There was no impact of HR on the surface- and time-averaged FFR_CT_ as ratio of average distal and proximal pressures, [7, 23]. However, there are some limitations in clinical use of indices invasively determined. Variability of hemodynamic factors such as heart rate, blood pressure, cardiac contractility, vascular compliance and the measurement itself may affect the measurement result.

Additionally, it should be noted that the numerical determination FFR_CT_ has also limitations.

The most important one is low image quality of CCTA and inaccurate fluid dynamics model for individual patients. In our study we have assumed of stroke volume independent of HR and lack of autoregulation.

An increased shear stress rate and turbulence at higher HR may contribute to a more frequent rupture of the atherosclerotic plaque. However, the mechanism of impact of HR on plague rupture is not yet fully understood [24].

### Stenosis degree

At a certain values of flow rate and stenosis degree, the flow is turbulent with distinct separation zones both upstream and downstream of stenosis. The transition from laminar to transient or turbulent flow causes an additional increase in the flow resistance, which is an unfavourable phenomenon that reduces the volumetric flow.

The significance of the stenosis may be assessed by comparison of the parameters directly related to the flow turbulence in the narrowed coronary vessel in a specific patient with those of modelled flow in a vessel without atherosclerosis.

Generally, an increase in degree of vessel narrowing increases in pressure drop and pressure drop coefficient. The surface average WSS is constant, independent from degree of stenosis.

This result is consistent with Murray’s law [25], according to which the WSS is constant, proportional to the velocity gradient and independent of the vessel radius. However, the flow disturbance induced by stenosis causes a deviation from this law, as a result of which the average value of WSS clearly depends on the degree of narrowing and active radius of the vessel. Above a certain value of stenosis, depending on the flow rate, there is a significant increase of WSS values (Fig. 4b).

The OSI is equal to zero for laminar flow in one direction. The maximum OSI value is 0.5 for purely oscillatory flow, without a resultant flow in a specific direction. The significance of low WSS stresses and a high OSI has been confirmed in the arteriosclerotic process [26]. However, the OSI does not provide a clear result if there are disturbances in the flow velocity. The index may be the same for both large velocity changes (strongly oscillatory flow) and small changes.

Therefore, the relative time of residence was introduced as a function of OSI and WSS averaged over time (AWSS). The analysis of this parameter also takes into account the impact of areas with low WSS index and high OSI [9] in order to more complete determine areas at risk of atherosclerosis.

Increasing the degree of stenosis results in an increase in mean and maximum OSI and RRT values. Maximum OSI value of 0.5 is achieved in a particular value of stenosis degree (~ 60%).

The local wall areas of maximum values of OSI and RRT, also increases. The areas of low WSS and high OSI and RRT occur downstream to the stenosis, but a “creeping” effect of these areas was observed which the size and location strongly depend on the degree of stenosis and artery geometry.

An axially non-symmetrical stenosis arranged on the internal wall of a bend or near a bifurcation introduces additional disturbances to the flow in the plane perpendicular to the axis of the vessel. Poisseuille’s formula implies that a 10% reduction in the radius of a vessel increases its resistance by 52%. In practice, however, the resistance increase is much lower due to interaction of autoregulatory mechanisms and the complex arrangement of vessels with parallel and serial connections.

Studies [7] are focused on the effect of fluctuations in HR and area stenosis on diagnostic indexes in a porcine model. They pointed out that, according to our study, FFR can be used for diagnose stenosis severity due to its independence from heart rate.

A clear impact on the FFR_CT_ stenosis occurs at severe degrees of stenosis. In the low degree of stenosis in the range of 10 to 60%, time and surface averaged FFR_CT_ changes by about 10% only. Therefore, so averaged FFR_CT_ seems not sensitive enough for the assessment of hemodynamic significance of stenosis in the 10 to 60% range. Additionally, a decrease in FFR_CT_ due to an increase in the degree of stenosis in one branch causes a slight increase in FFR in other branches, therefore the averaged FFR value at low values of constrictions is practically constant.

Even small changes in geometry can induce significant alterations of WSS [9, 20, 26]. Many articles supports the low or oscillatory shear theory. According to Kleinstreuer [27], areas with high time-averaged zero-dimensional WSS (> 1.5) as well as areas with low individual values (< 0.5) increase the probability of arteriosclerosis. Similarly, the probability of adhesion of pro-atherogenic participles and leukocytes to the endothelium may be higher in the event of a longer time of residence of blood near the arterial wall, a high OSI value [10], AWSS value lower than 0.4 Pa [20], and RRT above 10 Pa [28].

Authors [29] concluded that OSI and the relative residence time (RRT) could explain changes in plaque distribution as a result of changes in flow conditions and OSI-based risk factor was a better predictor than an AWSS-based one of subsequent stenosis.

Blood flow and disease localization was assessed at different stages in the disease process.

However, the conducted literature review revealed no reports concerning the impact of variable hemodynamic conditions (SV, CO, HR) on clinically indicated coronary index values. The current, most of the research present only the determination some hemodynamic parameters in a wide range of stenoses [30].

Blood flow and disease localization was assessed at different stages in the disease process. However, the conducted literature review revealed no reports concerning the impact of variable hemodynamic conditions (SV, CO, HR) on clinically indicated coronary indices values. The current, only a part of research present the numerical determination some hemodynamic parameters in a wide range of stenoses [30].

In clinical trials, measurements of coronary indices are made in anatomic and hemodynamic conditions specific for the patient, at the time of the medical examination. In turn, numerical simulations are based on patient-specific geometries using incomplete clinical data [5]. Therefore, our virtual numerical tests is carried out in variable hemodynamic conditions and in a wide range of stenosis, with the possibility of a task of varying initial/boundary conditions and with the possibility of numerical reconstruction of the geometry. In the case of a normal coronary vessel, without a narrowing, it is possible to model of the stenosis in places where the coronary hemodynamic indices reach the critical values. Inversely, in the case of stenotic coronary artery, on the basis 3D surface approximation of coronary wall, we can reconstruct the normal vessel without stenosis. Comparison of the results obtained from these two models (with and without stenosis) may allow for more accurate assessment of the significance and progression of stenosis.

The curves of relationship between flow condition and hemodynamic indices, contains the inflection points and/or local extrema reflecting the nature of the flow changes due to changes of vessel geometry and hemodynamic conditions. These characteristic points can help to assess the relevance of existing stenosis as well as allow you to determine the probability of atherosclerosis progression and location of places particularly exposed to the growth of atherosclerotic plaques in normal vessels. The same degree of stenosis may have different clinical significance. The hemodynamic significance of the coronary stenosis depend not only with severity of the stenosis but also anatomy of the coronary artery. In addition, stenosis in one branch has also effects on the hemodynamic parameters in side branches. For this reason, to non-invasive assessment of hemodynamic significance of coronary stenosis the diagnostic indexes should be studied under variable hemodynamic conditions, taking into account the impact of heart rate, cardiac output and degree stenosis on extreme values of hemodynamic indices, both near stenosis (locally) and in side branches (globally).

## Conclusion

There is significant variation in the coronary hemodynamic indices value caused by disturbed flow through narrowed vessels in relation to variable flow conditions and geometries. These variations may constitute a basis for assessing the hemodynamic significance of the stenosis in the coronary arteries. The upstream from the stenosis to point where extreme values of OSI or RRT occur may provide additional information to facilitate assessment of the hemodynamic significance of the stenosis.

Numerical studies conducted to evaluate of coronary flow in different conditions of flow rate and heart rate provide more complete information about the both location and extreme values of hemodynamic parameters. Computer modelling methods for coronary flow allow for the determination of critical values of the degree of stenosis and the dependence of coronary hemodynamic indices on variable flow conditions for the coronary artery in specific patients. However, for better assessment of the variability of indices and coronary stenosis severity both local (near stenosis) and global (in side branches) approach should be used.

Atherosclerotic lesions of arteries also occur in the left anterior descending artery in the vicinity of the origins of the septal arteries. They probably result from flow disturbances called the “milking-like effect” due to systolic compression of intramural septal branches [31]. A large part of a critical coronary events may also result from sudden vessel thrombosis due to atherosclerotic plaque rupture [32]. Thus, our next research will also take into account the mechanical properties of the coronary arteries and changes in tension due to external pressure on the vessel, as well as, the impact of mechanical stresses on atherosclerotic plaque rupture. To confirm the results obtained by virtual numerical tests, we need to analyse more clinical cases of coronary stenosis in future research.

## Abbreviations

AWSS: Time average
CCTA: Coronary Computed tomography angiography
CFD: Computational fluid dynamics
CO: Cardiac Output
CPD: Coefficient of pressure drop
CX: Left circumflex artery
FFR: Fractional flow
HR: Heart Rate
LAD: Left anterior descending artery
LM: Left main artery
RRT: Relative residence time
SV: Stroke Volume
WSS OSI: Oscillatory shear index
WSS: Wall shear stresses
ΔP: Pressure drop
